# The beginning and the end of SNARE‐induced membrane fusion

**DOI:** 10.1002/2211-5463.13447

**Published:** 2022-06-28

**Authors:** Delphine Mion, Louis Bunel, Paul Heo, Frédéric Pincet

**Affiliations:** ^1^ Laboratoire de Physique de l'École Normale Supérieure, ENS, Université PSL CNRS, Sorbonne Université, Université Paris Cité France; ^2^ Institute of Psychiatry and Neuroscience of Paris (IPNP) INSERM U1266 Paris France

**Keywords:** energy landscape, expansion, fusion pore, opening, SNAREpin initiation, vesicle

## Abstract

Membrane fusion is not a spontaneous process. Physiologically, the formation of coiled‐coil protein complexes, the SNAREpins, bridges the membrane of a vesicle and a target membrane, brings them in close contact, and provides the energy necessary for their fusion. In this review, we utilize results from *in vitro* experiments and simple physics and chemistry models to dissect the kinetics and energetics of the fusion process from the encounter of the two membranes to the full expansion of a fusion pore. We find three main energy barriers that oppose the fusion process: SNAREpin initiation, fusion pore opening, and expansion. SNAREpin initiation is inherent to the proteins and makes *in vitro* fusion kinetic experiments rather slow. The kinetics are physiologically accelerated by effectors. The energy barriers that precede pore opening and pore expansion can be overcome by several SNAREpins acting in concert.

## Graphical abstract

The assembly of SNAREpins provides the energy for fusing a vesicle with a target membrane. Fusion occurs in steps: membranes meet (i), SNAREs ‘find each other’ (ii) and initiate their assembly (iii). Next SNAREpins zipper and bring the membranes in close apposition (iv). Finally, a fusion pore opens (v) and expands (vi). Three SNAREpins are necessary to overcome the fusion and expansion energy barriers.

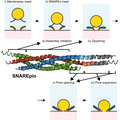

**Abbreviations**
FRET: Förster resonance energy transfer
MTC: multisubunit tethering complex
SFA: surface force apparatus
SNARE: soluble *N*‐ethylmaleimide–sensitive factor attachment protein receptor
SUV: small unilamellar vesicle
Syn1A: syntaxin 1A
TIRF: total internal reflection florescence microscopy
TMD: transmembrane domain
V_c_
: C‐terminal region of the SNARE domain of VAMP2 (typically residues 57–92)

Biological membranes reliably separate two aqueous regions and delineate the contours of cells and of the organelles they contain [1, 2]. Their integrity is ensured by their thin ∼ 3 nm hydrophobic core that prevents the crossing of any solute and sparsely allows water molecules to pass from one side to the other [3]. This spatial separation is indeed critical for them to separately accomplish their function [2]. Despite this individual specialization, organelles must work collectively. For instance, molecular exchanges must sometimes occur between them to share information and/or material. A major pathway for this molecular transport within or between cells is vesicular trafficking [4], which always follows the same steps. First, 50–200 nm vesicles containing selected cargo are formed from the membrane of the donor compartment. They travel to the target membrane where they fuse, thereby releasing both the encapsulated soluble cargo into the lumen and the membrane‐bound molecules in the membrane of the target compartment. This last key step of the transport process does not occur spontaneously. A high energy barrier, typically 25–30 k_B_T over a couple of nanometer displacement, needs to be overcome [5, 6, 7, 8, 9]. This high activation cost is not surprising because, to prevent untimely vesicle fusion, two very cohesive membranes must be actively, cooperatively, and simultaneously disrupted and merged.

It has been demonstrated more than a quarter of a century ago that the mechanical energy source of these mechanisms comes from proteins, the SNAREs [10, 11, 12]. These proteins form a complex between the two membranes, the SNAREpin. There is a whole family of SNAREpins [4]. They all contain a ‘SNARE domain’ characterized by four‐coiled alpha helices [13]. Each helix displays a heptad repeat, i.e., a hydrophobic residue every 3 and 4 alternating residues. In the coiled‐coil, the hydrophobic residues are aligned, forming ‘hydrophobic pocket’ or ‘hydrophobic layer’ [13, 14, 15]. Fifteen hydrophobic pockets in the SNARE domains are numbered from −7 at the membrane‐distal N‐terminus end to +8 at the membrane‐proximal C‐terminus end (Fig. 1A). The layer in the middle, referred to as layer 0, is hydrophilic and may help the correct register of the hydrophobic layers emanating from the four helices.

**Fig. 1:**
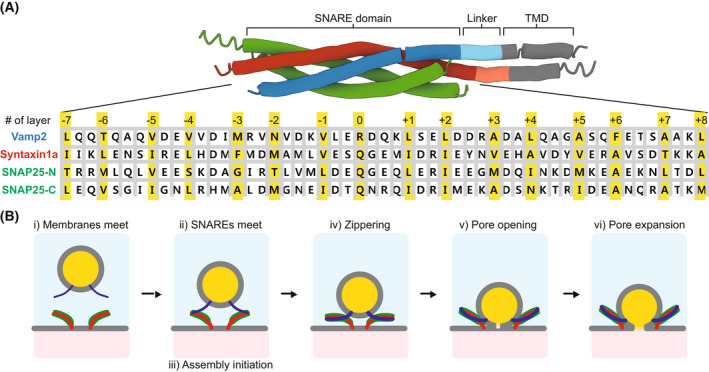
(A) SNAREpin molecular organization. The synaptic SNAREpin is held together by ‘SNARE domains’ coming from the v‐SNARE VAMP2 and from the t‐SNAREs Syntaxin1a and SNAP25. The 53 residues presented here for each SNARE domain are distributed in a heptad repeat fashion: Every 3 and 4 alternating residues are hydrophobic. The resulting hydrophobic pockets, highlighted in yellow and numbered from −7 at the N‐terminal end to +8 at the C‐terminal end, ensure the stability of the SNAREpin. The middle layer, referred to as layer 0, is actually hydrophilic. (B) These cartoons depict the six stages of SNARE‐induced fusion that occur when no other protein is involved, like in most *in vitro* experiments presented here but unlike evoked neurotransmitter release in which some of the stages are bypassed by chaperones.

An archetypal example is the SNAREpin responsible for the fusion of synaptic vesicles and the neuronal presynaptic plasma membrane. Since the synaptic SNAREpin is among the most studied and best characterized, we will focus on this specific one. This SNAREpin forms a four helical bundle composed of VAMP2 (also known as synaptobrevin) that contains a single cytosolic helix and a transmembrane domain (TMD) embedded in the synaptic vesicle and the binary complex made of syntaxin1a (Syn1A, one helix, and TMD) and SNAP25 (two helices separated by a linker containing cysteine clusters to conjugate to palmitic acids) on the presynaptic plasma membrane. Short linker domains (∼ 10 residues) connect the helix and TMD of Syn1a and VAMP2. The four helices of VAMP2, Syn1A, and SNAP25 represent the SNARE domains of the proteins.

The SNARE‐induced fusion process can be cut into six main stages (Fig. 1B). First, the membranes must meet (i). Then, SNAREs have to ‘find each other’ (ii) and initiate their assembly through their N‐terminal regions (iii). Next the SNAREpin zippers in an effort to bring the membranes in close apposition (iv). When the intermembrane distance is small enough, the membranes merge and a fusion pore opens (v) and subsequently expands (vi). This dissection of the fusion process is valid *in vitro* and *in vivo* when no other factor is involved which is not the case, for instance, in evoked neurotransmitter release where many steps are bypassed or facilitated by scaffold proteins; this will be briefly discussed.

Even though SNAREpins were proven to be a minimal, necessary, and sufficient machinery for fusion [12, 16], this breakthrough result was set in question because of the apparently slow kinetics. Fusion occurred on the timescale of dozens of minutes whereas the order of magnitude *in vivo* is seconds or minutes and can be as low as milliseconds for synaptic SNAREs [17, 18]. This surprising discrepancy is the starting point of our review: we will try to figure out and quantify the various kinetic and energetic hurdles during fusion induced by SNAREpins alone. We will not discuss stage (iv) that was exquisitely deciphered by optical tweezers [19, 20, 21]. The remaining stages will be split into two parts: towards SNAREpin assembly (stages i, ii, and iii) and fusion pore opening and expansion (stages v and vi). We will combine experimental observations and simple physics/chemistry models to show that SNAREpin initiation (iii), fusion pore opening (v), and subsequent expansion (vi) are the energetically limiting steps of the fusion process.

## Towards SNAREpin assembly: the beginning of the fusion process

**Fig. 2:**
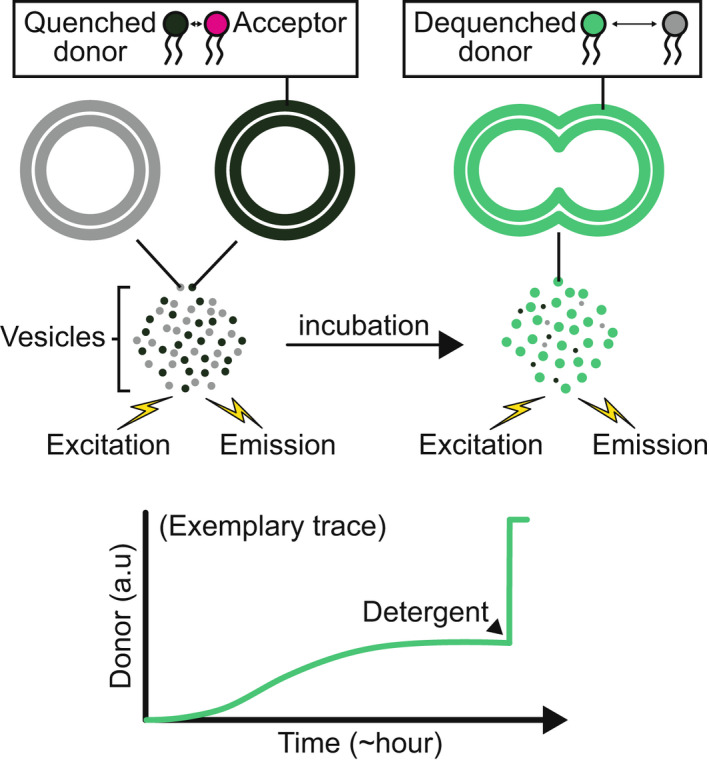
Standard lipid mixing bulk assay. Vesicles containing quenched fluorescent lipids and v‐SNAREs are mixed with an excess of nonfluorescent vesicles with t‐SNAREs (top left). Upon fusion of a fluorescent vesicle with a nonfluorescent one, dequenching occurs (top right). Hence, monitoring the fluorescence increase with time provides a direct measurement of the fusion process (bottom). To ensure that the fluorescence increase is indeed due to fusion, additional controls are needed, the most common of which being the content mixing depicted in Fig. 4A. The sudden rise at the end of the bottom cartoon depicts the addition of detergent, which maximizes the dequenching of the dyes and is used as a reference for analysis.

The historic observation that SNAREpins are the minimal machinery for fusion was performed with an *in vitro* ‘lipid mixing’ fusion assay (Fig. 2). In this first part, we will apply the common conditions (concentrations, vesicle dimensions, protein densities, …) used in this lipid mixing assay to quantitatively analyze and model the three stages that precede SNAREpin zippering.

### Getting in touch: meeting of the two membranes

**Fig. 3:**
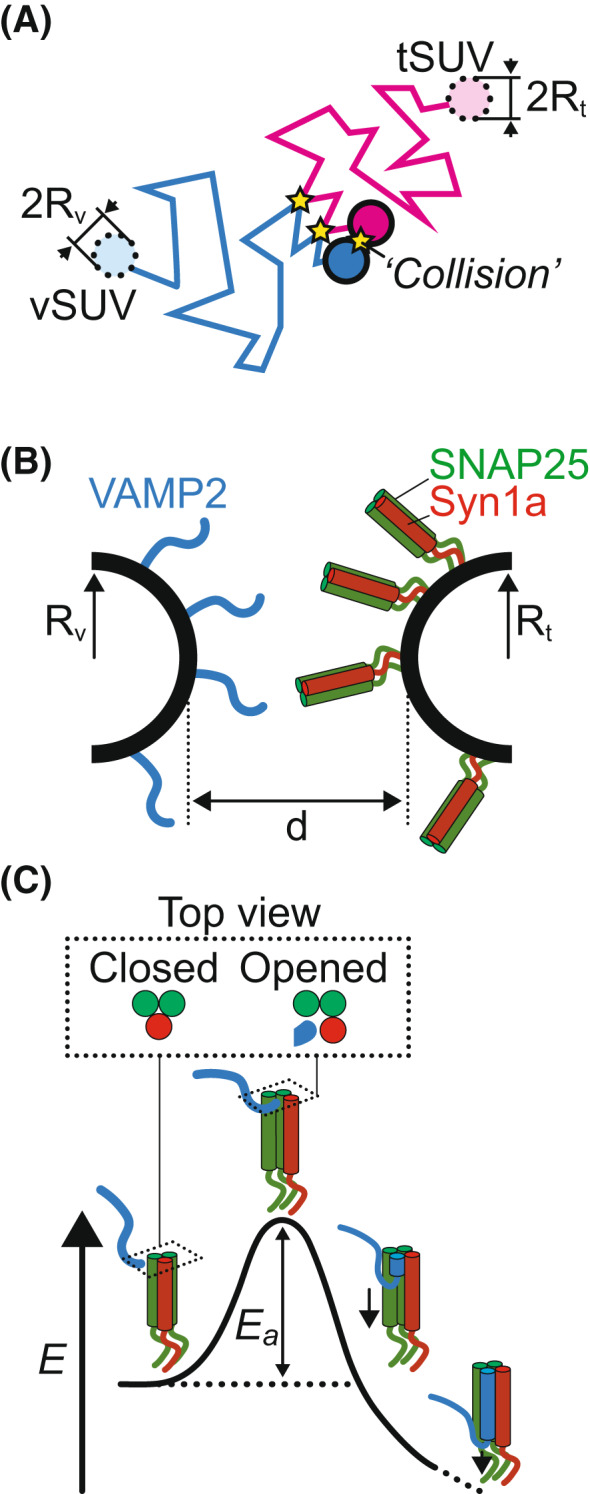
Initiation of SNAREpin assembly. (A) In the course of their movement, a vSUV and tSUV collide. After most collisions, they move away from each other (first two yellow stars), but sometimes, they stay bound through a SNAREpin. (B) For a SNAREpin to form, a v‐SNARE from the vSUV and a t‐SNARE from the tSUV must meet. (C) Meeting of the two SNAREpins is not sufficient. There initial assembly of the SNAREpin is limited by an energy barrier. In the lipid mixing bulk assay, this energy barrier comes from the need to disrupt the preassembled t‐SNAREs: Opening the 3‐helix bundle formed by their N‐terminal opens the groove for v‐SNARE to bind.

Prior to fusion, two free‐diffusing vesicles containing synaptic vesicle v‐SNAREs (vSUV) and target plasma membrane t‐SNAREs (tSUV) have to come in close proximity (Fig. 3A). The encounter rate depends on the vesicle concentration: the less concentrated, the fewer the collisions and consequently the slower the kinetics. This concentration effect can be quantitatively predicted by the theory of collision developed mainly by Smoluchowski [22]. This theory provides equations to compute the initial collision rate, ν, of vSUVs with tSUVs. If we note $R_{t}$ (resp. $R_{v}$) and $\rho_{t,\infty }$ (resp. $\rho_{v,\infty }$) the radius and the initial concentration in tSUVs (resp. vSUVs), the collision rate can be expressed as:

(1) $$\nu =\frac{2{\left(R_{t}+R_{v}\right)}^{2}k_{B}T\rho_{t,\infty }}{3\eta R_{t}R_{v}}$$

where $k_{B}$ is Boltzmann constant, T the temperature and η the viscosity of the solution (Eqn A7 in Appendix A). Under standard conditions of the lipid mixing assay, i.e., with 50 nm monodisperse SUVs mixed at 9 : 1 (mol%) of tSUV:vSUV for a total of 1 mm lipids, a vSUV will experience roughly 300 collisions with tSUV per second and there are about 2.10^14^ collisions between v‐ and tSUVs per second in 100 μL of solution. Under conditions with one VAMP2 per ∼ 100 lipids in vSUV and one t‐SNARE per ∼ 200 lipids in tSUV, it was found that the mean time for the first fusion event of a vSUV is typically 60 min [23, 24], which corresponds to ∼ 10^6^ collision events per vesicle. This is consistent with previous results that estimated only 1 fusion every 10^6^–10^7^ collision events [24, 25]. This very low yield of successful fusion per collision shows that one or several subsequent stages of the fusion process are slower than the meeting of the two membranes *in vitro*. Nevertheless, it is worth noting that, the collision rate being proportional to the vesicle densities, it can quickly decrease and become a slow step in diluted situations.

### Finding a mate: encounter of the cognate SNAREs

In the course of the collision of two vesicles, a pair of cognate t‐ and v‐SNAREs diffusing on the vesicle membranes will have to ‘find each other’ for fusion to proceed (Fig. 3B). This encounter of the two SNAREs can occur when the membranes are less than a certain distance d apart. The mean square displacement of a freely diffusing particle predicts the approximate time during which two vesicles, with a relative diffusion coefficient $D_{\mathrm{SUV}}$, remain less than a distance d apart:

(2) $$\tau_{\text{collision}}=\frac{d^{2}}{6D_{\mathrm{SUV}}}$$

Surface Forces Apparatus (SFA) measurements hinted that cognate SNAREs can find each other when the membranes are less than 8–15 nm apart [26, 27], which seems reasonable considering that the fully assembled complex, from N‐ to C‐terminal, has a size of 12 nm. For d= 15 nm and a typical value of 20 μm^2^·s^−1^ for $D_{\mathrm{SUV}}$, $\tau_{\text{collision}}$ boils down to a few microseconds. Random movement of a protein on an artificial membrane is characterized by a diffusion coefficient, $D_{\text{protein}}$. During the timescale $\tau_{\text{collision}}$, this leaves a SNARE time to explore an area

(3) $$a_{\exp}=4D_{\text{protein}}\tau_{\text{collision}}=\frac{2D_{\text{protein}}}{3D_{\mathrm{SUV}}}d^{2}$$

$D_{\text{protein}}$ was measured to be about 5 μm^2^·s^−1^ [28]. With these experimental values, $a_{\exp}$ scales as a few tens of nm^2^, which indicates that one SNARE covers the area of ∼ 100 lipids during a collision event. This threshold density, one SNARE per 100 lipids, is close to the standard SNARE density used in the lipid mixing assay and to the physiological density of v‐SNARE on a synaptic vesicle [29]. Below this threshold, our simple model predicts that the fusion rate should vary linearly with the density of SNAREs. A new analysis of a study that systematically varied SNARE densities on both v‐ and tSUVs [30] confirms this linear dependency (Appendix B). Above the threshold density, during the course of a collision, there is enough time for v‐SNAREs to completely cover the surface of the vSUV and meet an opposing t‐SNARE or any protein of similar dimension. Hence, at physiological SNARE densities, it seems that the encounter of the cognate SNAREs should occur systematically during the course of a collision.

### Overcoming timidity… : difficult initiation of SNAREpin assembly

In the lipid mixing assay, a v‐SNARE starts binding via its N‐terminal end on a well‐structured t‐SNARE (SNAP25 + Syn1) N‐terminal part (Fig. 3C, left) [31]. When the v‐ and t‐SNAREs meet, their very few N‐terminal residues weakly bind in a matter of at most a few seconds when membranes are 8 nm apart, as observed in the SFA [27]. However, it was also found that it takes up to half an hour to observe complete zippering. This long delay between initial contact was first attributed to the SFA geometry in which two macroscopic flat surfaces (∼ 1 cm^2^) are placed a few nanometers apart. This nonphysiological confinement of the proteins was assumed to slow down the process. It turns out that, considering the collision and fusion rates in the lipid mixing assay, this initial explanation of the long delay for the initiation of SNAREpin assembly is most likely incorrect. Indeed, it takes 1–10 million collisions for a vSUV to fuse with a tSUV under physiological concentrations of v‐SNARE in the lipid mixing assay. Each collision takes 10 μs and involves ∼ 10 v‐SNAREs that all have the opportunity to meet an opposing t‐SNARE. Assuming that there is no cooperativity between the SNAREpins and the first SNAREpin forms on average after a cumulated contact time for all SNAREs in play, a v‐SNARE needs to be in contact with a t‐SNARE for 100–1000 s on average. This delay is commensurate with the upper limit of 1800 s measured in the SFA.

Overall, this suggests that the initialization of SNAREpin zippering is the limiting step in the lipid mixing assay. From the 100–1000 s mean time required to start SNARE zippering, it is possible to estimate the activation energy of the process, $E_{a}$, using Kramers reaction‐rate theory [32, 33]. The mean time, τ, can be expressed as:

(4) $$\tau =\tau_{0}e^{\frac{E_{a}}{k_{B}T}}$$

For reactions that occur over a couple of nanometers, as is the case here, the prefactor time, $\tau_{0}$, is between 0.1 and 10 ns [33, 34], and $E_{a}$ would therefore be between 23 and 30 $k_{B}T$. This value is consistent with bulk measurements of the binding rate of v‐SNARE with t‐SNARE [35]. It was suggested that such a high energy value may be due to the necessity for the proteins to position and locally change structure to be able to bind [31, 35, 36]. A possible pathway for this structural change is suggested by experiments showing that in the 1:1 Syntaxin:SNAP25 t‐SNARE complex, i.e., the physiological stoichiometry, the N‐terminal portion forms a three‐helix coiled coil while the C‐terminal region remains frayed [36, 37]. The N‐terminal coiled coil would need to be opened for the v and t‐SNAREs to bind [36, 37, 38]. It has also been long known that structuring the C‐terminal part of the t‐SNARE by prebinding it with the soluble cognate v‐SNARE region, V_c_, accelerates SNAREpin assembly [31, 39]. A putative explanation to conciliate these observations is that V_c_‐binding structures the four‐helix bundle at the C‐terminal part of the SNARE domain, and this structure propagates in the N‐terminal region of the t‐SNARE domain, thereby opening the groove where v‐SNARE can directly bind. *In vitro* experiments suggest that this structural remodeling reduces the activation energy for SNAREpin initiation to 8 $k_{B}T$ [35], which would make the SNAREpin assembly extremely fast (0.3–30 μs according to Eqn 4).

### …with the help of chaperones

From the minimal initial assembly models presented above, two main hurdles to achieve fusion can be identified: vesicle – target membrane meeting and SNAREpin zippering initiation. These limitations may look like they would create difficulties in physiology by slowing down the fusion process, just as it was observed in the lipid mixing assay. Actually, they can be switched on or off by effectors and appear as assets used by cells and organelles to control and induce SNAREpin formation [40].

To bring and maintain the vesicles at a distance compatible with SNAREpin formation, 10–20 nm, long tethers are used. They include the long banana‐shaped protein Munc13 that can form complexes with proteins from the RIM and Rab families [41, 42, 43, 44, 45, 46, 47, 48]. By extending these tethers away from the target membrane surface near calcium channels, these complexes are able to capture vesicles and position them at the location where calcium will enter during neurotransmission. A pool of vesicles can thus be permanently docked, thereby overpassing the difficulty for the vesicles to meet the target membrane [49]. This description corresponds to the synaptic SNAREpins. For other SNAREpins, other macromolecular complexes are used to tether the vesicles. They are often referred to as Multisubunit Tethering Complexes or MTCs [50, 51].

Switching off the energy barrier for initial SNAREpin assembly requires another effector. This function is achieved by Sec1/Munc18‐like proteins [52, 53, 54, 55, 56, 57]. To better understand the activation role of Munc18, a point needs to be clarified regarding t‐SNAREs. In most *in vitro* experiments, the t‐SNARE complex made of Syn1A and SNAP25 is preassembled. This is not the case *in vivo*. Hence, the activation energy for SNAREpin initiation presented above cannot quantitatively represent the physiological reality: for example, there is no need to open a groove in the t‐SNARE for v‐SNARE to bind. However, in neurons, the N‐terminal part of the Syn1A SNARE domain forms a four‐helix bundle with the so‐called Habc N‐terminal of Syn1A [58, 59, 60]. This coiled‐coil needs to be disrupted to allow SNAP25 and VAMP2 binding. This disruption of the protein complex requires energy that will be a barrier to initial SNAREpin assembly. To our knowledge, the actual value of this energy barrier has not been measured but, because it entails disrupting more bonds than the opening of a groove *in vitro*, it is likely to be larger than the 23 and 30 $k_{B}T$. At the molecular level, Munc13 is needed to open the Habc domain [53], Munc18 can grab the N‐terminal of both Syn1A and VAMP2 SNARE domains [61, 62] and bring them together [63, 64]. SNAP25 binds to Munc13, which chaperones its assembly with Syn1A and VAMP2 to initiate SNAREpin formation [65].

Finally, the membrane distribution of the t‐SNARE may help chaperones to accelerate the initial SNAREpin assembly by increasing encounter probability between cognate SNAREs. For instance, Syn1A is known to form *in vivo* microdomains of different sizes in equilibrium with freely diffusing proteins. Super‐resolution techniques hint at clusters of diameter 50–80 nm with 30–90 copies of Syn1A, colocalizing with SNAP25 clusters having at least a similar number of copies [66, 67, 68, 69, 70]. The evidence hence suggests the existence of domains with very high concentrations in t‐SNAREs, scaling as tens of thousands of complexes per μm^2^, which probably improves the speed of the docking and priming process. The size, composition, structure, and organization of the clusters are not yet fully understood but might be controlled by lipid composition, protein–protein interactions [66, 67], the inclusion in an active zone [68], and the presence of a primed vesicle [69, 70]. These possibilities of modulating clusters could as well provide more control over fusion.

## Pore opening and expansion: the end of the fusion process

In this second part, we will focus on the final action of the SNAREpins: the fusion process itself, i.e., the actual merging of the two membranes into a single entity. As the SNAREpin zippers, the apposed membranes come in close proximity. When the remaining water layer between them is 1–2 nm, depending on the membrane composition [8, 9], they are destabilized and a fusion pore opens. This short intermembrane separation at fusion suggests that only the C‐terminal regions of the SNAREs, probably beyond layer +3, may play an active part in the actual fusion process. This hypothesis is consistent with experimental observations [71, 72].

At the molecular level, the destabilization of the membranes towards the formation of the fusion pore is a complex process that has been the focus of many studies [73, 74, 75, 76, 77, 78, 79, 80, 81, 82, 83, 84, 85, 86]. SNAREpins may actually influence this molecular pathway, favor the formation of intermediate lipid and protein arrangements [87, 88], and affect the nature of the pore (see Box 1 for related discussion). Each fusion event will go through a different molecular pathway since hundreds of molecules are involved and the geometry of lipids will favor some fusion pathways over others [6, 94, 95, 96, 97]. To circumvent this variability inherent to complex systems, we will envision the fusion process as a single reaction with a global activation energy barrier that needs to be passed to open the fusion pore. The main reason for this approach is that, experimentally, fusion is usually demonstrated by the actual opening of a fusion pore and not by the intermediate states. In any case, SNAREpins lead to the same final result: the formation of an extended fusion pore.

Box 1 **Nature of the fusion pore: Lipids, proteins, both?**

Understanding the molecular nature of the fusion pore is a prerequisite to understand quantitatively how the fusion pore opens and expands. This is a difficult task because at 1 to 10 nanometer scale, molecules are very dynamic, and the timescale for movement is dozens of ns [89]. This fast movement of the molecules always needs to be kept in mind: there is no such thing as a constant nature of a fusion pore. In any case, we will try to identify the molecular regions that are the most likely to be decorating the rim of the pore. Since the pore is aqueous it will always be energetically more favorable to have hydrophilic motives exposed to the inside of the pore. However, in lipid bilayers, it is well documented that hydrophobic parts are frequently facing the aqueous region. In the same way, there is no doubt that lipid chains and hydrophobic residues from the SNARE transmembrane domains can transiently be exposed at the rim of the pore.

The best picture of the typical molecular nature of the fusion pore is probably obtained by molecular dynamics simulations. They show that the pore is mainly decorated by polar heads of lipids and C‐terminal regions of the SNAREs [90]. Experimental observation suggests that the transmembrane domains can also be in contact with aqueous phases [91]. It remains unclear whether they are in direct contact with the aqueous pore or with inverted micelles that may form during the fusion process [92].

In summary, the pore appears to be mainly delineated by polar heads of lipids with a scarce presence of protein residues, primarily coming from the C‐terminal region of the SNAREs and possibly also from the transmembrane domain [93].

In this part devoted to the formation and expansion of the fusion pore, we will first describe the different types of observations, present the current view of pore opening and expansion, and model the energetics involved in each step.

### How to probe the fusion pore

Two main types of experimental measurements are performed: optical and electrical.

**Fig. 4:**
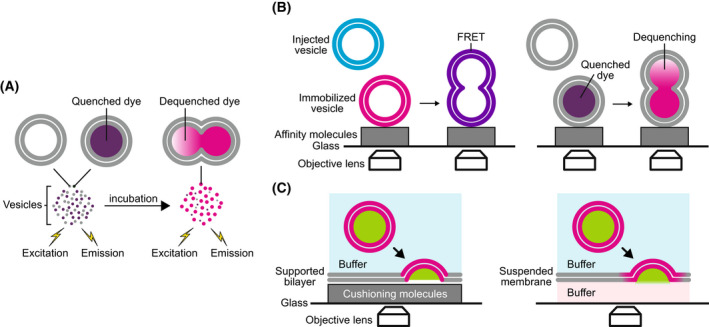
Optical assays (in addition to the lipid mixing assay presented in Fig. 2). (A) In the content release bulk assay, dyes are quenched in the vSUV. Upon fusion, these dyes are diluted and their fluorescence increases. Monitoring this increase in time provides a direct quantification of the content released during fusion. (B) Single vesicles immobilized on a surface can be monitored by total internal reflection fluorescence microscopy (TIRF). The fusion of a vesicle with cognate SNAREs can be observed either by Forster resonance energy transfer (FRET) for lipid mixing, left, or by dequenching of encapsulated dyes for content release (right). (C) Fusion of a vesicle with a flat membrane, supported (left) or suspended (right), can be observed by TIRF. Dequenching of membrane‐bound or encapsulated dyes provides a direct observation of single fusion event at the level of lipid mixing and content release, respectively.

Optical observations using fluorescence dequenching can be achieved in bulk or at the single fusion event level. Quenched fluorescent dyes are placed in the vesicle, either bound to the membrane as in the lipid mixing assay presented in the first part (Fig. 2) or in the lumen, referred to as ‘content mixing’ assays (Fig. 4) [98, 99, 100, 101, 102, 103, 104, 105, 106, 107, 108]. Upon fusion, the dyes diffuse away from the vesicle and their release is observed by the resulting increase in fluorescence due to the dequenching. The main limitation of lipid mixing assays is that they do not directly account for the opening of a fusion pore. For instance, a hemifusion state in which only the external leaflets of the two membranes have merged may be mistakenly confused with fusion. Lipid mixing also provides limited information on the fusion pore kinetics because the dyes are released extremely fast, typically in ms for a 1 nm diameter pore (see Appendix C). Conversely, the release of encapsulated fluorescent dyes through a fusion pore occurs on a slower time scale because the pore first needs to expand. The main difficulty of content release assays is to verify that the cargo does not diffuse away from the vesicle through leaks induced by the mechanical action of surface tension or by chemical modification of the membrane properties. Ideally, both ‘lipid mixing’ and ‘content release’ assays should be performed in parallel to ensure the validity of the results.

**Fig. 5:**
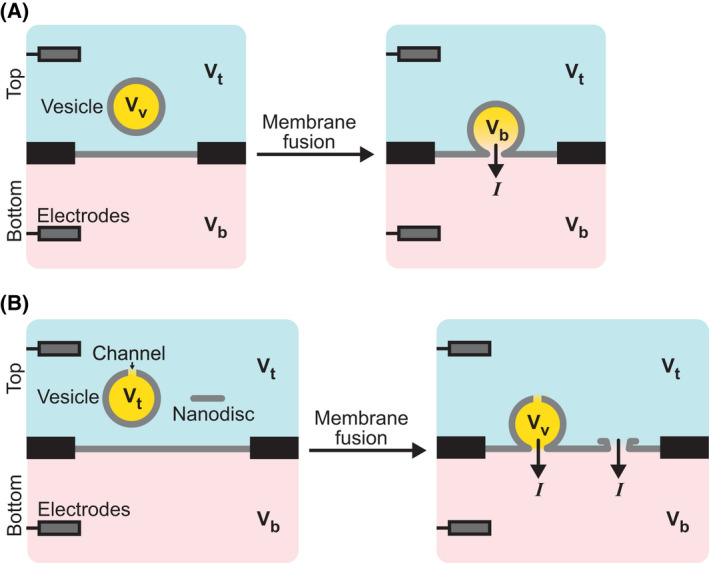
Electrical assays. The fusion of a vesicle with a suspended membrane can be observed by placing two electrodes on either side of the membrane. (A) Impedance measurements. Upon fusion, the membrane of the vesicle is incorporated in the suspended membrane, which increases the capacitance. Also, initially, the potential in the lumen of the vesicle is different from the potentials on either side of the suspended membrane. Hence, upon fusion, there is a transient current to equilibrate the potentials of the vesicle, V_v_, and the lower side of the membrane, V_b_ (right panel). This current is due to ions that flow through the pore and therefore provides a direct measure of the pore kinetics. (B) Conductance measurements. Because the current in the impedance measurement is transient, the kinetics of the pore can only be measured over a short period of time (~ 1 ms). To obtain longer kinetics, a constant voltage can be applied between the two sides of the suspended membrane. Using vesicles with embedded channels, thus at the same potential as the top side (V_t_), or nanodiscs, ions flow continuously when the fusion pore opens; the whole kinetics of pore opening and expansion can then be monitored.

Electrical observations can also be used to monitor the kinetics of the fusion pore [109, 110]. They require to place at least one electrode on each side of the target membrane. In theory, monitoring the impedance of the vesicle/target membrane system during the fusion process allows the simultaneous characterization of the pore kinetics and the vesicle size by measuring the conductance and capacitance, respectively (Fig. 5A). In reality, the conductance only provides transient information because the voltage difference between both sides of the fusion pore quickly vanishes to zero. This issue can be resolved by imposing a permanent voltage between the two sides of the target membrane and placing the lumen of the vesicle in electrolytic contact with the vesicle exterior (Fig. 5B). Electrolytic contact can be achieved by either adding channels in the vesicle membrane [110] or replacing the vesicle with a small membrane patch, called nanodisc [103, 104, 105].

Finally, in the last years, with the increasing computational strength and the theoretical progress in the field, molecular dynamics simulations have proven to be a more insightful method to numerically probe the structural and functional properties of biological systems. Molecular dynamics simulations, by providing unique information on molecular remodeling and arrangement during fusion, nicely complement experimental observations.

We will now discuss the two main steps in the fusion process: the nucleation, i.e., the opening of a pore, and its subsequent growth.

### How to seed a fusion pore

Before discussing SNARE‐induced fusion pore opening, it is important to understand the dimensions and energies involved. Here, we will present a model assuming that membrane interactions involved in the fusion process are purely associated with the physical and chemical properties of lipid bilayers; proteins may actually alter these interactions but are unlikely to significantly change the orders of magnitude (see Box 1). The analogy noticed almost 50 years ago between the fusion process and the transition from lamellar phases to other phases, e.g., hexagonal or rhombohedral phases [7, 8, 111], provides quantitative insights. Merging the membranes and opening a fusion pore necessitates overcoming the sharp short‐range hydration/protrusion forces between membranes. These repulsive surface forces, $F_{R}$, decay exponentially with the separation distance, d:

(5) $$F_{R}\left(d\right)=P_{0}e^{-\frac{d}{\lambda }}$$

with a characteristic length, λ, of a few Angstroms and a prefactor, $P_{0}$, of about 100 atm [7, 8]. This explains why merging the membranes and opening a fusion pore is energetically costly, ∼ 25 $k_{B}T$ [5, 6]. Since this energy must be provided over a very short distance, typically 1 nm [7, 8], the average force is 100 pN. Assuming that the pore opens at 100 atm pressure, this force should be applied to an area of 10 nm^2^, which is occupied by 15 lipids. Hence, the initial opening of a fusion pore probably involves ∼ 100 lipids when accounting for both leaflets.

A 25 $k_{B}T$ energy barrier is sufficient to prevent spontaneous fusion. Indeed, just as in the first part for the initiation of the SNAREpin, we can use Kramers reaction‐rate theory (Eqn 4, [32, 33]), to estimate the waiting time, $\tau_{w}$, before thermal fluctuations provide enough energy for passing the fusion barrier, $E_{b}$:

(6) $$\tau_{w}=\tau_{0}\;\exp\left(\frac{E_{b}}{k_{B}T}\right)$$

Equation (6) indicates that the waiting time is in the minute scale for $E_{b}=25\;k_{B}T$, which predicts that fusion will not spontaneously occur on an experimentally relevant time scale for neurotransmission, highlighting the physiological need for SNAREpins. Here, we will provide a simple model describing N SNAREpins temporarily clamped in a partly assembled state and simultaneously released, approximately mimicking the role of the calcium sensor Synaptotagmin‐1. In this model, the acceleration of the fusion process by SNAREs can be quantitatively estimated by calculating the duration of two distinct phases in the SNAREpin: approaching the membranes and the actual opening of the fusion pore. First, the SNAREpins must reduce the vesicle—target membrane distance from their initial separation to the minimum of the energy landscape before the fusion barrier, i.e., 2 to 3 nm. This is achieved by the pulling force applied by each SNAREpin, $F_{p}$. Because the system is overdamped, the speed of the vesicle, v, is driven by the drag force, i.e., the Stokes force, that opposes the N SNAREpins pulling force:

(7) $$v=\frac{NF_{p}}{3\mathrm{\pi d\eta}}$$

where η is the viscosity of the surrounding aqueous medium and d the vesicle diameter. Hence, the time, $\tau_{t}$, to travel a distance is:

(8) $$\tau_{t}\left(N\right)=\frac{3\text{πldη}}{NF_{p}}$$

where l is the total displacement of the vesicle. Once the vesicle has reached the minimum of the energy landscape, it faces the fusion barrier that must be overcome by thermal fluctuations for fusion to occur. Because the SNAREpins are pulling on the membranes they reduce the height of the fusion barrier. Hence, using Kramers’ reaction‐rate theory, the waiting time for N SNAREpins becomes:

(9) $$\tau_{w}\left(N\right)=\tau_{0}\;\exp\left(\frac{E_{b}-\mathrm{N\delta e}}{k_{B}T}\right)$$

where δe is the energy reduction in the fusion barrier due to a single SNAREpin.

The fusion time to bring the vesicle from their initial separation distance to contact and subsequent fusion $\tau_{f}\left(N\right)$ is the sum of the travel and the waiting time:

(10) $$\tau_{f}\left(N\right)=\tau_{t}\left(N\right)+\tau_{w}\left(N\right)=\frac{3\text{πldη}}{NF_{p}}+\tau_{0}\;\exp\left(\frac{E_{b}-\mathrm{N\delta e}}{k_{B}T}\right)$$

**Fig. 6:**
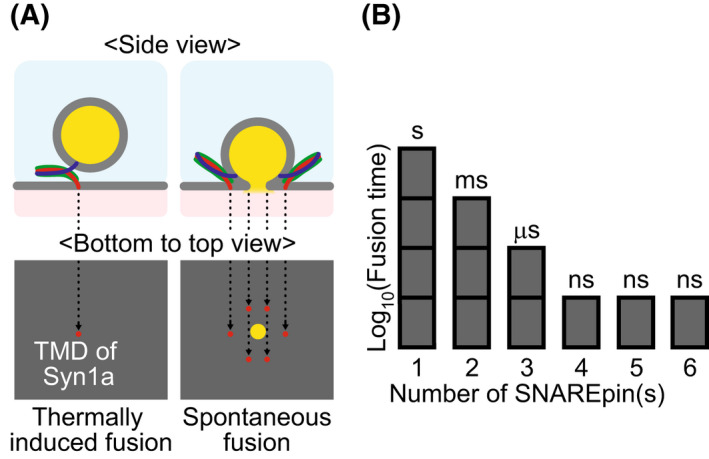
Fusion time. (A) When only one SNAREpin is involved (left), there remains an activation energy barrier for fusion. Hence, thermal fluctuations will provide the final stroke for fusion pore opening. The mean time for fusion in that case is ~ 1 s. When 6 SNAREpins are acting simultaneously (right), the fusion barrier vanishes and fusion is spontaneous. The only remaining delay is the travel time of the vesicle to a 2 nm distance to the target membrane, typically a few ns. (B) Variation of the fusion time with the number of SNAREpins based on the parameters indicated in Appendix D. An additional membrane merger time may need to be added and become the dominant term for more than 3 SNAREpins.

Two regimes are predicted for $\tau_{f}\left(N\right)$ (Fig. 6A). In the first regime, at low N, the waiting time is limiting. Then, the fusion time decays exponentially with the number of SNAREpins. In the second regime, at higher N, the travel time is limiting and $\tau_{f}\left(N\right)$ is inversely proportional to N. Using the values in Appendix D, Eqn (8) predicts a threshold value of *N* = 4 SNAREpins for which the fusion time is dozens of ns, i.e., extremely fast (Fig. 6B). These predictions are in quantitative agreement with the experimental observations suggesting that it takes ∼ 1 s for a single SNAREpin to drive fusion [112, 113]. Fig. 6B also implies that three or more SNAREpins must act simultaneously to achieve neurotransmitter release in less than 1 ms in synaptic transmission. The prediction of this simple model on the number of SNAREpins is consistent with experimental observations [114, 115] and molecular dynamic simulations showing once the SNARE domains are almost fully zippered, the membranes are in such close apposition that the polar headgroups of the outer lipid leaflets are dehydrated to a level allowing fusion [116].

The simple model we present here suggests a monotonic decrease in the fusion time with the number of SNAREpins. Intriguingly, several models suggest that there is an optimum SNAREpin number for fast fusion because using too many SNAREpins in the contact area of the vesicle and the target membrane would actually slow down the fusion process [116, 117]. Two reasons for the existence of an optimum number have been proposed. First, steric repulsions increase the equilibrium intermembrane docking distance impeding efficient fusion. Molecular dynamics simulations predict a shift of the membrane separation from 2 to 3 nm when varying the number of active SNAREpins from 7 to 13 [116]. Second, a mechanical model shows that the SNAREpins, which are not sufficiently zippered provide a force opposing fusion; the predicted optimal number of SNAREpins before this effect becomes dominant is 3 to 7 [117]. There is no experimental proof yet of the existence of such an optimum number of SNAREpins.

**Fig. 7:**
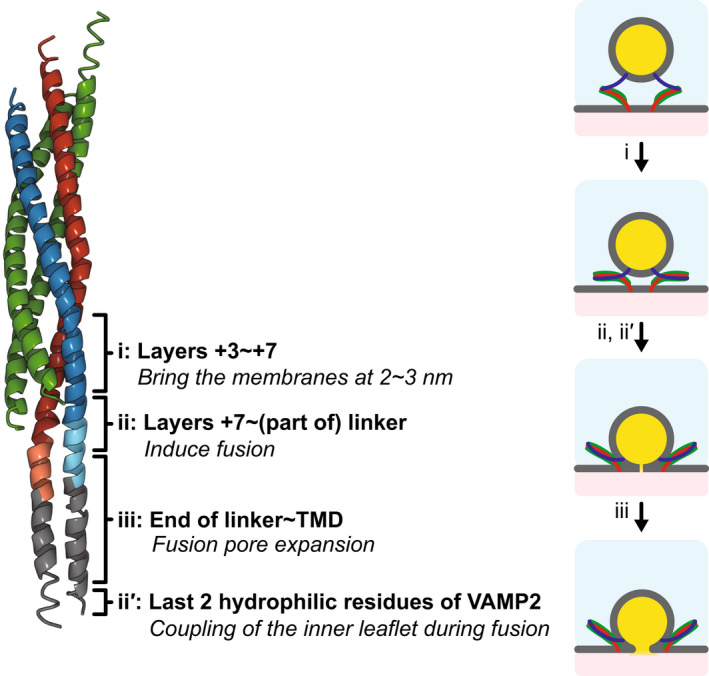
Role of the various parts of the C‐terminal end of the SNAREpin for fusion pore opening and expansion.

For fusion to actually occur, the zippering force applied by the SNAREpins to the apposing membranes needs to be transmitted by the linker and transmembrane domains (Fig. 7). To test the actual role of these domains, experiments and molecular dynamics simulations have been performed with specific mutations, deletions, or substitutions with lipid chains [15, 90, 118, 119]. The assembly of the linker and transmembrane domains into coiled coils seems to provide energy to help pore opening and possibly subsequent expansion. However, there is an open question on the structure and rigidity of the linker domain that condition the efficiency of the force transmission.

The last element of the SNAREs that plays a significant part in the nucleation process is the very C‐terminal end of VAMP2 [120]. VAMP2 has 2 hydrophilic uncharged residues after the transmembrane domains that are preserved across species, usually Ser‐Thr or sometimes Ser‐Ser. Several studies show that they play an important role in pore opening by inducing the deformation of the bilayer around the C‐terminal leading to the nucleation of the pore by forcing the rearrangement of lipids. Because the vesicle has a high positive curvature in contrast to the nascent fusion pore characterized by a high negative curvature, a dramatic change in curvature occurs on the vesicle side. The two hydrophilic residues provide leverage for this transition. Intriguingly, Syn1A ends with the transmembrane domain without any subsequent hydrophilic residue. The curvature changes on the target membrane side are not as drastic and the strong anchorage of the t‐SNARE in the hydrophobic core through Syn1A and SNAP25 seems to be sufficient to ensure optimal fusion [71, 121].

### How to grow a fusion pore

**Fig. 8:**
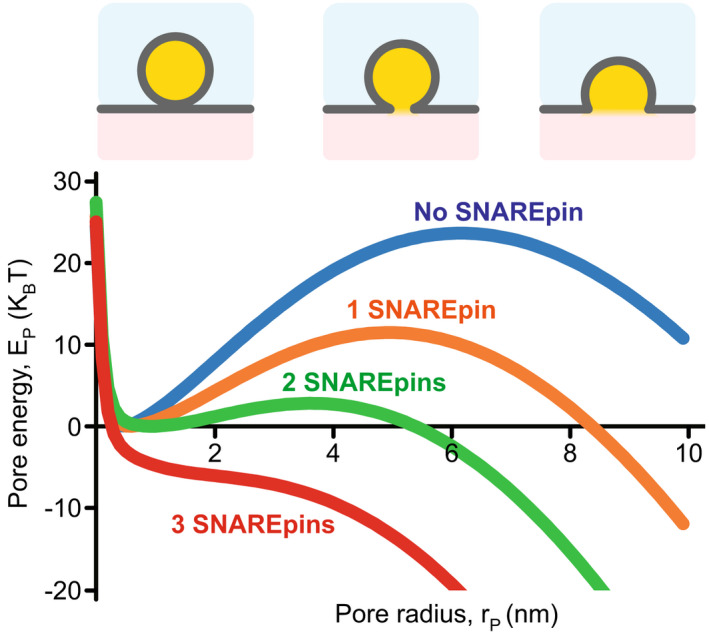
Expansion barrier. Opening the fusion pore is not sufficient to ensure full fusion. An expansion barrier due to curvature energies prevents the growth of the nascent fusion pore. Examples of energy landscapes of the pore expansion without SNAREpin (blue) and upon the action of one (orange), two (green), or three (red) SNAREpins. For these predictions, the vesicle radius, bending modulus, height of the vesicle distance, membrane thickness, and SNAREpin contribution were set at 25 nm, 10 $k_{B}T$, 2 nm, 5 nm, and 10 pN, respectively. 10 pN means each SANREpin provides ~ 2.5 $k_{B}T$ per nm increase in the pore radius. The quantitative details of the model used to obtain these landscapes are described in Appendix E.

Opening a fusion pore is not sufficient to ensure full fusion. The importance of the subsequent expansion of the pore must not be underestimated because it is not a spontaneous process and also requires some energy. Expansion of the nascent fusion pore is associated with the energetically costly extension of the highly curved rim. Those curvatures will be continuously reduced as the pore extends (see Appendix E for explanations). Using a simple model based on curvature energy and crude torus‐like geometry, there is a threshold pore diameter corresponding to an expansion barrier (Fig. 8). If the pore expands above this threshold diameter, it spontaneously expands. Conversely, if there is not enough energy to pass the barrier, the pore ultimately reseals. Resealing is not a straightforward process either since, just like for opening the fusion pore, the two membranes that form the rim of the pore must merge to form fully distinct lumens. In this situation, the pore is trapped in a transiently open state and will eventually reseal when thermal fluctuations provide enough energy to overcome this resistance to resealing.

Considering each SNAREpin provides a constant force towards the expansion of the fusion pore, the energy landscape with one, two, or three SNAREpins can be computed in the crude torus‐pore model. Using the energy landscape for pore expansion resulting from this model and the typical force applied by SNAREpins, 3 SNAREpins would start spontaneously expanding the pore (Fig. 8 and Appendix E). Several types of *in vitro* experiments with nanodiscs, vesicles, and suspended membranes have investigated the effect of the number of SNAREpins on the nascent fusion pore [103, 110, 114, 115]. These studies consistently suggest that one or two SNAREpins are indeed able to open a fusion pore but cannot expand beyond the expansion barrier, making the fusion pore transient. The average apparent diameter of a fusion pore induced by a single (resp. two) SNAREpin(s) seems to be in the range of 0.3–0.4 nm (resp. 0.8–0.9 nm) [110]. These transient pores reseal after a few 100 ms when the SNAREpins run out of energy, i.e., when the transmembrane domains are fully zipped.

After fusion pore opening, the SNARE domains and at least part of the linker domains are already assembled. Hence, the energy for expanding the pore is expected to come from the zipping of the transmembrane domains. This is indeed what is experimentally observed. When the transmembrane domains are replaced by lipid chains or other noninteracting transmembrane domains, cargo release is reduced to the level induced by one or two SNAREpins [115].

### How to catalyze pore opening and expansion *in vivo*?

We saw that the formation of an expanded fusion pore is energetically opposed at two stages of the process: the fusion and the expansion barriers. Intriguingly, while a couple of SNAREpins are not sufficient to bypass these barriers, a handful of simultaneously acting SNAREpins provides enough energy to make them both disappear, making the fusion process spontaneous. Hence, for cells to precisely control the time of fusion, several SNAREpins must be synchronized. This synchronicity is primarily achieved by several copies of the calcium sensors Synaptotagmin‐1 that clamp a few SNAREpins in a partly zipped state and synchronously release them upon calcium entry. Another potential protagonist of this synchronization on the synaptic vesicle, synaptophysin, forms hexameric structures necessary to make the synaptic vesicle functional and is able to bind VAMP2 [122, 123, 124, 125]. This organization may regulate the number of v‐SNAREs presented to the target membrane [126]. In this cryoelectron tomography study, it was proposed that each protein complex of the hexameric structure contains one partly assembled SNAREpin with VAMP2 emanating from the Synaptophysin:VAMP2 complex. To match this hexameric structure on the plasma membrane side, it has been proposed that Munc13, possibly helped by Synaptotagmin‐1, oligomerizes in a ring‐like structure, facilitating the assembly of exactly 6 SNAREpins [127]. The perfect matching of the symmetry between the two membranes is an appealing solution to guarantee that the optimum number of SNAREpins are acting together when fusion is triggered. However, these mechanisms still remain to be proven both structurally and functionally.

## Conclusion

The overall SNARE‐induced fusion process is considerably slowed down by three main energy barriers: initial assembly of the SNAREpin, fusion pore opening, and pore expansion. Initial assembly occurs at the very N‐terminal part of the SNARE domains and requires structural changes in the t‐SNARE that are energetically costly. To open and expand the fusion pore, each part of the SNAREpins from layer +3/+4 of the SNARE domain to the very C‐terminal plays a specific part. Zipping of the SNAREpin from layer +3 to +7 is responsible for bringing the two membranes into molecular contact. Zipping of layers +7, +8, and possibly part of the linker domain provides the energy for overcoming the fusion barrier. It is likely that 5 SNAREpins or more are necessary to make the fusion barrier disappear although thermal fluctuations are sufficient to overcome it in less than 100 μs when 3 or more SNAREpins are acting together. The linkers transmit the zipping force to optimize the action of the SNARE domains and provide the final energy stroke to open the fusion pore. The C‐terminal hydrophilic residues of VAMP2 reinforce this force transmission by facilitating the deformation of the vesicle membrane. The zipping of part of the linker domains and of the transmembrane domains might be in charge of pore expansion. Experimental results and models suggest that the expansion barrier disappears when 3 SNAREpins or more are simultaneously zipping.

## Conflict of interest

The authors declare no conflict of interest.

## Author contributions

DM has worked on all models and the part related to the initiation of the SNAREpin. LB focused on the pore opening and the nature of the pore. PH was involved in the transient pore studies, pore expansion, and the figure design. FP worked on the model and supervised the research. All authors wrote the manuscript.

## Figures and Tables

**Fig. 9:**
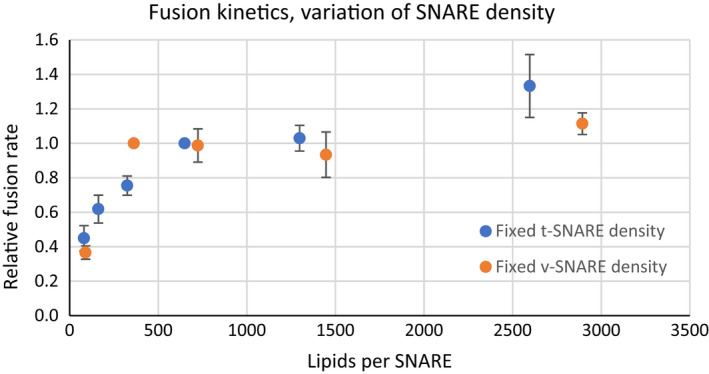
Mean normalized fusion rates, $p_{v}$ and $p_{t}$, defined in the text, are presented against the lipid to protein ratio. The curves display a plateau above approximately 500 lipids per SNARE for both v‐ and t‐SNARE, suggesting that the SNARE exhibit no cooperativity and that their contributions are additive.

**Fig. 10:**
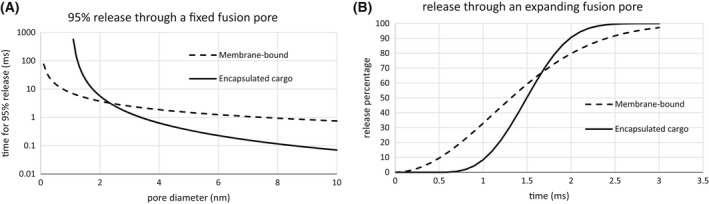
Release of molecules through the fusion pore. (A) The characteristic times of release of membrane‐bound molecules (dashed line) and encapsulated cargo (full line) in nm are computed from Eqns (C6,C8), and the resulting 95% release times of the molecules are plotted against the pore diameter in nm. (B) Percentage of release of membrane‐bound and encapsulated molecules through a pore expanding at 1 nm·ms^−1^. The curves presented here are for a 50 nm vesicle, a 10 nm long pore and a cargo of 0.5 nm hydrodynamic radius. The diffusion coefficient of the membrane‐bound molecules was chosen at 10 μm^2^·s^−1^, which is standard for a lipid.

**Fig. 11:**
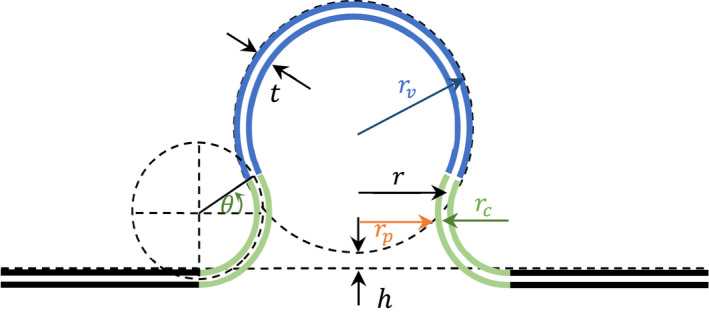
A vesicle (blue) is fusing with a flat membrane (black). The fusion pore is assumed to be a partial circular torus (green).

**Fig. 12:**
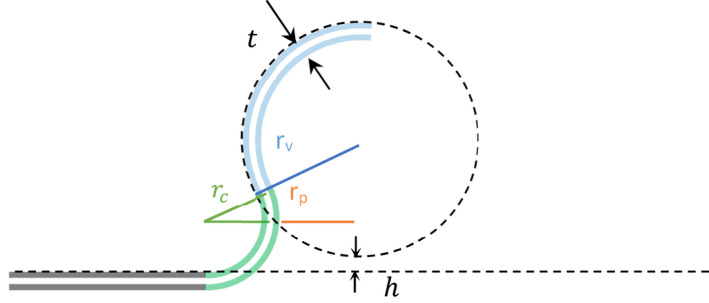
The expression of $r_{c}$ is obtained through the triangle presented here. The sides of the triangle are, respectively, $\;r_{v}-\left(r_{c}-t/2-h\right)$, $r_{p}+r_{c}+\frac{t}{2}$, and $r_{v}+r_{c}-\frac{t}{2}$ for the purple, green/orange, and green/blue sides.

## Appendix A Rate of collision of vesicles/nanodiscs in a lipid mixing assay

The rate of collision of vesicles or nanodiscs in the bulk can be computed with the standard Smoluchowski approach [22].

We consider two types of vesicles/nanodiscs:

• Those containing v‐SNAREs, named ‘v‐particle’ in the following, of hydrodynamic radius $R_{v}$, bulk concentration $\rho_{v,\infty }$, and diffusion coefficient $D_{v}$.
• Those containing t‐SNAREs, named ‘t‐particle’ in the following, of hydrodynamic radius $R_{t}$, bulk concentration $\rho_{t,\infty }$, and diffusion coefficient $D_{t}$.

Let us fix the coordinate system on the center of a v‐particle. We want to know the flux of t‐particles colliding with our v‐particle because of diffusion processes. The spatiotemporal profile of the concentration in t‐particles $\rho_{t,\infty }$ is given by Fick's second law:

(A1)
$$\frac{\partial \rho_{t}}{\partial t}=D\Delta \rho_{t}$$

whereD is the diffusion coefficient of *t*‐particles in the referential of *v*, which can be shown to be $D=D_{t}+D_{v}$.

Stokes‐Einstein's equation then gives us

(A2)
$$D=\frac{k_{B}T\left(R_{t}+R_{v}\right)}{6\pi \eta R_{t}R_{v}}$$

with η the dynamic viscosity of the solution, which is approximately equal to that of water.

In a steady‐state regime, Eqn (A1) boils down to the Laplace equation:

(A3)
$$\Delta \rho_{t}=0$$

which can be solved in spherical coordinates.

Assuming that, upon a collision, there is no aggregation (fusion or bouncing back are rapid events) the boundary condition around the *v*‐particle is:

(A4)
$$\rho_{t}\left(R_{t}+R_{v}\right)=0$$

The spatial concentration of the t‐particle at a distance r from the considered v‐particle can then be obtained from Eqns (A3,A4):

(A5)
$${\rho_{t}}_{{|}_{r>R_{t}+R_{v}}}=\rho_{t,\infty }\left(1-\frac{R_{t}+R_{v}}{r}\right)$$

The flux of *t*‐particles coming in collision with our *v*‐particle can then be deduced from Fick's first law:

(A6)
$$J=-D{\frac{d\rho_{t}}{\mathrm{dr}}}_{{|}_{R_{t}+R_{v}}}=\frac{k_{B}T\rho_{t,\infty }}{6\pi \eta R_{t}R_{v}}$$

By integrating the flux over the sphere of radius *R*_*t*_ + *R*_*v*_, we finally get the following collision rate:

(A7)
$$\nu =\frac{2{\left(R_{t}+R_{v}\right)}^{2}k_{B}T\rho_{t,\infty }}{3\eta R_{t}R_{v}}$$

What is remarkable here is that the collision rate only depends on the ratio between the two radii. In particular, if the two objects have the same radius:

(A8)
$$\nu =\frac{8k_{B}{\mathrm{T\rho}}_{t,\infty }}{3\eta }$$

To find orders of magnitude that are consistent with what is usually done experimentally, we will take v‐ and t‐vesicles of monodisperse radius 25 nm, each of them with a final lipid concentration of 1 mm [12, 24, 30]. The molecular area of a lipid is typically 0.65 nm^2^ [8]. Thus, the number of lipids per SUV can be estimated to be 20 000. The resulting molar concentration in vesicles is 50 nm, i.e., the t‐vesicle concentration is $\rho_{t,\infty }=2.5.10^{19}$ vesicles·m^−3^, which yields ν ∼ 300 collisions·s^−1^. Given an initial slope of the corrected dequenching curve ∼ 1·4000 s^−1^ for vesicle‐vesicle, this means less than 1 collision out of 1 million is successful [24, 25].

## Appendix B SNARE additivity and cooperativity in the lipid mixing bulk assay

According to Eqn (3), during a collision, a SNARE covers the area occupied by ∼ 100 lipids. Assuming there is no cooperativity such as oligomerization between SNAREs, when the lipid to protein ratio is significantly larger than 100, each SNARE can be considered independent of the others. With this assumption, the probability that a SNAREpin starts assembling from a specific vSUV will vary linearly with the concentration of VAMP2 in the vSUV and with the concentration of t‐SNARE in the tSUV. Hence, the fusion rate in the lipid mixing bulk fusion assay, $\nu_{f}$, should be inversely proportional to the lipid to protein ratios in both types of SUVs:

(B1)
$$\nu_{f}\propto \frac{\nu_{\mathrm{ref}}}{r_{v}r_{t}}$$

Where $r_{v}$ (resp. $r_{t}$) is the lipid to protein ratio in the vSUV (resp. tSUV) and $\nu_{\mathrm{ref}}$ a reference fusion rate.

The assumption that the SNAREs behave independently of each other and the existence of threshold lipid to protein ratios can be tested by comparing the fusion rates at various values of $r_{v}$ and $r_{t}$. The fusion kinetics has previously been systematically measured at different, accurately measured lipid to SNARE ratios varying from ∼ 80 to ∼ 3000 lipids per outward‐facing SNARE [30]. We reanalyzed the data and considered the initial kinetics are well represented by the percentage increase in fluorescence at 80 min. This approach underestimates the kinetics at high protein density because the fusion rate will go down as more vSUV fuse with tSUV but is the most accurate in the lower concentrations.

To check Eqn (B1), we took ${r_{v}}_{\mathrm{ref}}=$ 649 lipids per VAMP2 as a reference and, for each $r_{v}$, we averaged $\frac{\nu_{f}\left(r_{v}\right)r_{v}}{\nu_{f}\left({r_{v}}_{\mathrm{ref}}\right){r_{v}}_{\mathrm{ref}}}$ over all tested $r_{t}$. We calculated the same parameter for the t‐SNARE taking ${r_{v}}_{\mathrm{ref}}$ = 362 lipids per t‐SNARE. According to our assumptions that there is no cooperativity of the SNARE in the lipid fusion bulk assay and that the SNARE contributions are additive below a concentration threshold, the resulting parameters, $p_{v}$ and $p_{t}$, should be equal to 1 above a certain lipid to protein ratio. Figure B1 confirms this prediction suggesting that the SNAREs do not exhibit any cooperativity and have additive contributions to fusion under these experimental conditions.

## Appendix C Kinetics of lipid mixing and content release

In this Appendix, we will consider the fusion of a vesicle with an infinitely large target membrane. The extracellular medium will also be considered infinitely large. The vesicle membrane initially contains membrane‐bound molecules (resp. encapsulated cargo) at a concentration $c_{m0}$ (resp. $c_{c0}$). The fusion pore is assumed to have a fixed radius, $r_{p}$, and a length, $L_{p}$. We will estimate the concentration of membrane‐bound molecules, $c_{m}$, and encapsulated cargo, $c_{c}$, remaining in the vesicle in time. At any time, the concentration of membrane molecules (resp. cargo) initially in the vesicle that diffused to the target membrane (resp. extracellular medium) is zero. Hence, the membrane molecule and cargo gradients in the pore can be written as $\frac{c_{m}}{L_{p}}$ and $\frac{c_{c}}{L_{p}}$, respectively. Using Fick's first law, the variation of $c_{m}$ in time can be written as:

(C1)
$$\frac{\partial c_{m}\left(t\right)}{\partial t}=-\frac{2\pi r_{p}}{4\pi r_{v}^{2}}D_{m}\frac{c_{m}\left(t\right)}{L_{p}}=-\frac{r_{p}D_{m}}{2r_{v}^{2}L_{p}}c_{m}\left(t\right)$$

where $D_{m}$ is the diffusion coefficient of the molecules and $r_{v}$ the vesicle radius.

The situation is slightly more complex in volume because the pore radius needs to be larger than the hydration radius of the cargo, $r_{c}$. Hence, the effective pore radius is $\left(r-r_{c}\right)$. Fick's first law leads to:

(C2)
$$\frac{\partial c_{c}\left(t\right)}{\partial t}=-\frac{3\pi {\left(r_{p}-r_{c}\right)}^{2}}{4\pi r_{v}^{3}}D_{c}\frac{c_{c}\left(t\right)}{L_{p}}=-\frac{3{\left(r_{p}-r_{c}\right)}^{2}D_{c}}{4r_{v}^{3}L_{p}}c_{c}\left(t\right)$$

where $D_{c}$ is the diffusion coefficient of the cargo, which can be estimated from the Stokes‐Einstein equation:

(C3)
$$D_{c}=\frac{k_{B}T}{6\pi r_{c}\eta }$$

Hence Eqn (C2) can be rewritten:

(C4)
$$\frac{\partial c_{c}\left(t\right)}{\partial t}=-\frac{{\left(r_{p}-r_{c}\right)}^{2}k_{B}T}{8\pi r_{v}^{3}L_{p}r_{c}\eta }c_{c}\left(t\right)$$

For a pore of fixed radius, Eqns (C1,C4) can be readily integrated:

(C5)
$$c_{m}\left(t\right)=c_{m0}e^{-\frac{t}{\tau_{m}}}$$

With the following expression for the characteristic time, $\tau_{m}$:

(C6)
$$\tau_{m}=\frac{2r_{v}^{2}L_{p}}{r_{p}D_{m}}$$

And:

(C7a)
$$c_{c}\left(t\right)=c_{c0}e^{\left(-\frac{t}{\tau_{c}}\right)}\;\text{if}\;r_{p}>r_{c}$$

(C7b)
$$c_{c}\left(t\right)=c_{c0}\;\text{if}\;r_{p}<r_{c}$$

With the following expression for the characteristic time, $\tau_{c}$:

(C8)
$$\tau_{c}=\frac{8\pi r_{v}^{3}L_{p}r_{c}\eta }{{\left(r_{p}-r_{c}\right)}^{2}k_{B}T}$$

95% of the molecules (membrane‐bound or encapsulated) are released after three characteristic times. The typical examples for the release time of 95% of the molecules presented in Fig. 10 show that encapsulated cargos will be released much slower than membrane‐bound molecules for fusion pores up to ∼ 1 nm in diameter but will be faster released for larger pores. Hence, for an efficient cargo release during fusion, the pore needs to expand beyond a few nm. The membrane‐bound molecule will be released in ms even with a small pore.

Physiologically, several SNAREs act on the membrane. The fusion pore radius increases in time with a speed v almost constant, typically 1 nm·ms^−1^ [109]. In that case, Eqns (C1,C4) become:

(C9a)
$$\frac{\partial c_{m}\left(t\right)}{\partial t}=-\frac{{\mathrm{vD}}_{m}}{2r_{v}^{2}L_{p}}tc_{m}\left(t\right)$$

(C9b)
$$\frac{\partial c_{c}\left(t\right)}{\partial t}=-\frac{k_{B}T}{8\pi r_{v}^{3}L_{p}r_{c}\eta }{\left(\mathrm{vt}-r_{c}\right)}^{2}c_{c}\left(t\right)$$

which can be integrated:

(C10a)
$$c_{m}\left(t\right)=c_{m0}e^{-\frac{{\mathrm{vD}}_{m}}{4r_{v}^{2}L_{p}}t^{2}}$$

(C10b)
$$c_{c}\left(t\right)=c_{c0}e^{-\frac{k_{B}T}{24\mathrm{\pi v}r_{v}^{3}L_{p}r_{c}\eta }\left[{\left(\mathrm{vt}-r_{c}\right)}^{3}\right]},\text{for}\;t>\frac{r_{c}}{v}$$

(C10b)
$$c_{c}\left(t\right)=c_{c0},\text{for}\;t<\frac{r_{c}}{v}$$

Figure C1B shows the release of membrane‐bound and encapsulated cargo. Expanding the fusion pore ensures a fast and complete release of both types of contents in a couple of milliseconds, which is critical for neurotransmission.

## Appendix D Time for fusion

We will choose an energy reduction in the fusion barrier due to a single SNAREpin, δe, of 6 $k_{B}T$, which is similar to that previously predicted [117]. For $E_{b}=25\;k_{B}T$, 5 SNAREpins or more will completely abolish the fusion barrier. The force applied by a single SNAREpin is of the order of 30 pN in this range of intermembrane distance (∼ 30 $k_{B}T$ energy gain over a 4 nm displacement [20]). With these values, the travel time of the vesicle from a 5 nm separation to 2 nm before fusion, the decreased fusion barrier, the waiting time, and the fusion time can be calculated from Eqns (8,9,10). The results are presented in Table 1. The fusion time is plotted in Fig. 6B.

**Table 1:** A vesicle initially located 5 nm from the target membrane is pulled by N SNAREpins, brought in contact and fusion occurs subsequently. The travel time, reduction in the fusion barrier, waiting time, and fusion time are indicated for 1 to 6 SNAREpins.

N number of acting SNAREpins	$\tau_{t}$ travel time	$E_{b}-\mathrm{N\delta e}$ fusion barrier ($k_{B}T$)	$\tau_{w}$ waiting time	$\tau_{f}$ fusion time
1	50 ns	19	0.2–2 s	0.2–2 s
2	24 ns	13	0.4–4 ms	0.4–4 ms
3	16 ns	7	1–10 μs	1–10 μs
4	12 ns	1	3–30 ns	15–42 ns
5	9 ns	0	0	9 ns
6	8 ns	0	0	8 ns

## Appendix E Energy landscape of the fusion pore expansion

**Preliminary description of the model**

Let us consider a fusion pore between a vesicle of external radius $r_{v}$ and a flat membrane that opens as the two objects are at a distance h that we will assume constant during the pore expansion process (Fig. E1). The thickness of the lipid bilayers is noted t. The lipid bilayer will be considered as a continuous and differentiable (i.e., ‘smooth’) curve, which is a simplifying but also bold assumption at this scale.

We will focus on the case in which the pore has a circular tore‐like geometry. The radius (taken up to the middle of the bilayer) of the small circle forming the tore by rotation is noted $r_{c}$ and increases as the fusion pore expands. The portion of the tore spans from an angle $-\frac{\pi }{2}$ to a maximal angle called $\theta_{m}$, which will decrease. We will call θ the variable describing the portion of the circle between $-\frac{\pi }{2}$ and $\theta_{m}$.

We can also choose a cylindrical coordinate system to describe the shape of the pore with the radial coordinate denoted r that describes the radius of the pore at a certain height, taken up to between the monolayers. The radius of the pore taken up to its rim will be noted, $r_{p}$; it is the minimum value of $r-\frac{t}{2}$.Given our parameters, we have that:

(E1)
$$r=r_{p}+\frac{t}{2}+r_{c}\left(1-\cos\left(\theta \right)\right)$$

$r_{c}$ can be obtained from the Pythagorean theorem (see Fig. 12):

(E2)
$$r_{c}=2r_{v}-r_{p}+h-t/2-\sqrt{2\left(2r_{v}^{2}-\mathrm{ht}+hr_{v}-2r_{v}t-hr_{p}-2r_{v}r_{p}\right)}$$

Finally, we can compute $\theta_{m}$ through:

(E3a)
$$\cos\left(\theta_{m}\right)=\frac{r_{p}+\frac{t}{2}+r_{c}}{r_{v}+r_{c}-\frac{t}{2}}$$

(E3b)
$$\sin\left(\theta_{m}\right)=\frac{r_{v}-r_{c}+\frac{t}{2}+h}{r_{v}+r_{c}-\frac{t}{2}}$$

**Computation of the curvature energy**

We will assume here that curvature is the sole driving force. Hypothesizing that the membranes are spontaneously flat, the curvature energy of the system is $E_{c}=\kappa /2\iint_{V+T}{\left\langle c\right\rangle }^{2}\mathrm{dS}$, where K is the membrane bending modulus, <c> is the mean curvature at the considered point, V is the surface of the vesicle and T that of the partial circular torus that forms the pore.

***Curvature energy of the fusing vesicle***

The curvature energy of the fusing vesicle is that of the full vesicle minus that of the spherical cap of surface $S=2\pi {\left(r_{v}-\frac{t}{2}\right)}^{2}\left(1-\sin\left(\theta_{m}\right)\right)$ that disappeared because of fusion. The curvature for the vesicle being constant equal to 2/(rv‐t/2) at any point of the vesicle, the total curvature energy of the vesicle is:

(E4)
$$E_{v}=\frac{K}{2}\frac{4}{{\left(r_{v}-\frac{t}{2}\right)}^{2}}\left[4\pi {\left(r_{v}-\frac{t}{2}\right)}^{2}-S\;\right]=4\mathrm{K\pi}\left[1+\sin\left(\theta_{m}\right)\right]$$

***Curvature energy of the tore***

The curvature on the torus is given by:

(E5)
$$\left\langle c\right\rangle =-\frac{\cos\left(\theta \right)}{r}+\frac{1}{r_{c}\;}$$

The elementary surface dS is given by: $\mathrm{dS}=2\mathrm{\pi r}\;r_{c}\mathrm{d\theta}$, so that the torus curvature energy is:

(E6)
$$E_{t}=\frac{\mathrm{\pi K}}{r_{c}\;}\int_{-\frac{\pi }{2}}^{\theta_{m}}\frac{{\left(r-r_{c}\;\cos\left(\theta \right)\right)}^{2}}{r}\mathrm{d\theta}$$

By substituting *r* by its expression and noting $a≔\frac{r_{p}+\frac{t}{2}+r_{c}}{r_{c}}>1$, we get:

(E7)
$$\begin{array}{rcl} E_{t} & = & \mathrm{\pi K}\int_{-\frac{\pi }{2}}^{\theta_{m}}\frac{{\left(a-2\;\cos\left(\theta \right)\right)}^{2}}{a-\cos\left(\theta \right)}\mathrm{d\theta}\\ & = & \mathrm{\pi K}\int_{-\frac{\pi }{2}}^{\theta_{m}}\left(-4\cos\left(\theta \right)+\frac{a^{2}}{a-\cos\left(\theta \right)}\right)\mathrm{d\theta} \end{array}$$

Substituting $u=\sqrt{\frac{a+1\;}{a-1}}\;\tan\left(\theta /2\right)$ leads to $\int_{-\frac{\pi }{2}}^{\theta_{m}}\frac{a^{2}\;}{a-\cos\left(\theta \right)}\mathrm{d\theta}=\frac{2a^{2}\;}{\sqrt{a^{2}-1}}\int_{-\sqrt{\frac{a+1\;}{a-1}}}^{\sqrt{\frac{a+1\;}{a-1}}\tan\left(\theta_{m}/2\right)}\frac{1\;}{1+u^{2}}\mathrm{du}$

which provides the final expression of the torus curvature energy:

(E8)
$$E_{t}=\begin{array}{l} \mathrm{\pi K}[-4\left(\sin\left(\theta_{m}\right)+1\right)+\frac{2a^{2}}{\sqrt{a^{2}–1}}(\arctan\left(\sqrt{\frac{a+1}{a-1}}\tan\left(\frac{\theta_{m}}{2}\right)\right)\\ +\arctan\left(\sqrt{\frac{a+1}{a-1}}\right))] \end{array}$$

Total curvature energy

The sum of Eqns (E4,E8) provides the total curvature energy:

(E9)
$$E_{c}=\begin{array}{l} \frac{2{\mathrm{\pi Ka}}^{2}}{\sqrt{a^{2}–1}}[\arctan\left(\sqrt{\frac{a+1}{a-1}}\tan\left(\frac{\theta_{m}}{2}\right)\right)\\ +\arctan\left(\sqrt{\frac{a+1}{a-1}}\right)]. \end{array}$$

It is worth noticing that the vesicle curvature energy is exactly compensated by one of the terms of the energy of the pore, which is probably not innocuous.

**Energy landscape of the pore**

In addition to the curvature energy, an energy barrier for resealing needs to be added to the pore energy landscape. This barrier is due to the merging of the rim upon pore closure. Hence, it should resemble that of the fusion barrier. A typical energy landscape of a fusion pore based on E9 is presented in Fig. 8. An expansion barrier as high and much larger than the resealing barrier is clearly observed. This resistance to expansion arises from the high curvature energies involved in the process.

The next step is to add SNAREpins and observe how they affect the pore expansion energy landscape.

Assuming each SNAREpin contributes with a force f, the energy landscape of the pore upon the action of N SNAREpins is reduced by:

(E10)
$$E_{s}=-\mathrm{Nf}r_{p}$$

Using reasonable values for all parameters predicts that the pore will spontaneously expand only when there are three SNAREpins acting simultaneously (Fig. 8). The local minima for one and two SNAREpins are located at a pore radius of 0.6 and 0.9 nm, respectively. Overall, our model seems consistent with what was put forward experimentally [110].

However, those theoretical results should be taken very cautiously for the crude approximations made are to be challenged, including:

• Continuum approach for the bilayer whereas scales are that of lipids.
• The distance between membrane and vesicle taken fixed, whereas it probably decreases over time.
• The vesicle shape assumed to remain spherical whereas it likely deforms during the process.
• The circular torus‐like geometry does not represent all possible membrane shapes.
